# Synthesis and Optimization of the Docetaxel-Loaded and Durvalumab-Targeted Human Serum Albumin Nanoparticles, In Vitro Characterization on Triple-Negative Breast Cancer Cells

**DOI:** 10.1021/acsomega.3c02682

**Published:** 2023-07-13

**Authors:** Fatma Yurt, Derya Özel, Ayça Tunçel, Ozde Gokbayrak, Safiye Aktas

**Affiliations:** † Department of Nuclear Applications, Institute of Nuclear Science, Ege University, Bornova, 35100 Izmir, Turkey; ‡ Department of Basic Oncology, Institute of Oncology, DokuzEylül University, 35340 Izmir, Turkey

## Abstract

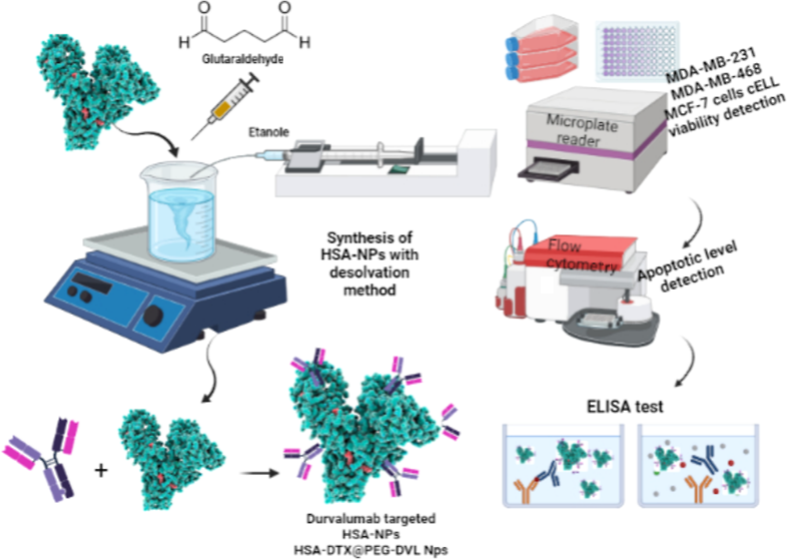

Triple-negative breast cancer (TNBC) tends to behave more aggressively compared to other breast cancer subtypes due to the lack of receptors and its limited targeting therapy. In recent years, nanotechnology advancement has led to the development of various nanoparticle platforms for the targeted treatment of cancers. Especially, HSA-NPs have specific advantages such as biocompatibility, adjustable size during production, and relatively easy synthesis. In this study, HSA-NPs were encapsulated with docetaxel (DTX) and functionalized with polyethylene glycol (PEG), also becoming a targeting nanoplatform modified with durvalumab (DVL), and the whole nanostructure was well characterized. Subsequently, drug release studies and various *in vitro* cell culture studies such as determining the cytotoxicity and apoptotic levels of the nanoplatforms and PD-L1 using ELISA test were conducted on MDA-MB-468, MDA-MB-231, and MCF-7 cells. According to the results, HSA-DTX@PEG-DVL NPs showed better cytotoxicity compared to DTX in all the three cell lines. In addition, it was observed that the HSA-DTX@PEG-DVL NPs did not lead the cells to late apoptosis but were effective in the early apoptotic stage. Moreover, the ELISA data showed a significantly induced PD-L1 expression due to the presence of DVL in the nanostructure, which indicates that DVL antibodies successfully bind to the HSA-DTX@PEG-DVL nanostructure.

## 1 Introduction

Triple-negative breast cancer (TNBC) is a kind of breast cancer and frequently is the leading cause of death among women. Because TNBC has limited targeting therapy due to negative estrogen, progesterone, and Her-2/neu receptors and is a more aggressive type of breast cancer tumor with a faster growth rate, higher risk of metastasis, and recurrence risk compared to other breast cancer subtypes. Conventional therapies such as chemotherapy, surgery, and radiation therapy are the primary systemic treatment strategies used in the most common treatments of TNBC.^1^ Generally, chemotherapy and radiotherapy are the next approaches used for the treatment of remaining cancer cells after the surgical removal of tumors due to the recurrence risk of the tumor site.^2^ However, current chemotherapeutic drugs have not been convincing because of killing of healthy cells and the toxicity caused to the patients. On the other hand, discovering new anticancer drugs is a time-consuming and costly process. Therefore, the current research focuses on increasing the anticancer potential of the existing anticancer drugs through the design of new drug delivery systems. Accordingly, it is critical to discover new, effective, and safe methods as an alternative to the existing methods used in the treatment of TNBC.^3^

In recent years, nanotechnology advancement has led to the development of various nanodrug formulations for the treatment of TNBC. Thus, they have provided an opportunity to achieve individualized treatment.^4^ Nowadays, many anticancer drugs are designed in the form of nanoparticles, which are more advantageous than using these drugs.^5^ Especially, drug delivery with HSA NPs (human serum albumin nanoparticles) has specific advantages such as biocompatibility, reproducible synthesis, adjustable size during production, and relatively easy synthesis.^6−11^ In a physiological setting, many metabolic compounds, therapeutic molecules, and therapeutic drugs, which include hydrophobic as well as hydrophilic drugs, are transported by HSA through their size, abundance, and binding sites available on the three-dimensional structure.^8,12,13^ These properties have led to improved tumor targeting studies due to their ability to passive targeting through the enhanced permeation and retention (EPR) effect. Normally, macromolecules through the structure of tumor vasculature lack effective lymphatic drainage in tumor tissues.^14^ However, nanocarriers can accumulate in tumors through the EPR effect by utilizing the structural features of the tumor environment.^15^ On the other hand, the physical stability, surface characteristics, and size of the nanoparticles highly influence the retention of these drugs within the body. Nanoparticles with a smaller size could enter the lymphatic capillaries and undergo clearance, whereas nanoparticles with the size larger than 250 nm were identified by macrophages and removed by the reticuloendothelial system. However, EPR-mediated passive targeting of nanoparticles suffers from difficulties in intracellular drug delivery.^5^ On the contrary, the distribution of the NPs could also be provided for adjustment by active targeting.^16^ Surface modification of NPs with targeting molecules is crucial for anticancer drugs, which can enhance drug concentration in the targeted cells or normal tissues causing to decrease the drug concentration and their side effects. Active tumor targeting is achieved by conjugating synthesized nanoparticles to the specific molecules that bind to overexpressed receptors on the surface of the targeted tumor cells. These targeting molecules can be categorized as proteins, nucleic acids such as aptamers, and monoclonal antibodies (mAbs).^5^

In our previous works, the preparation of docetaxel (DTX)-loaded HSA NPs to design a tumor-targeted drug delivery system based on the HSA conjugation strategy has been reported.^17^ Since DTX is a very toxic anticancer agent with low water solubility, to overcome these side effects and improve the antitumor efficacy of DTX, this time it was encapsulated in HSA NPs produced by a desolvation method and achieved an alternative system for DTX delivery. This study is aimed at using HSA-NPs as the carrier of DTX and to design effective nanoplatforms in targeted TNBC cells with durvalumab (DVL) effective in the PD-L1 pathway (HSA-DTX@PEG-DVL).

Programmed cell death receptor-1/programmed death ligand-1 (PD-1-/PD-L1)-based cancer immunotherapy is a popular and promising approach for many types of cancer treatment, especially in TNBC. Using these antibodies to block PD-1 and PD-L1 has shown promising outcomes over conventional methods in advanced cancers.^18^ PD-L1, also called the B7 homolog 1 (B7–H1), is expressed not only in T cells but also in other immune cells and different types of cancer. PD-L1 is a cell surface protein expressed on activated APC, T and B lymphocytes, and other cells and is the natural ligand of PD-L2 and PD-1. Interaction of PD-L1 on PD-1-activated T cells results in immunosuppression, and as time progresses, the immune escape mechanism of the tumor is activated.^18^ There are many different antibodies targeting PD-L1. These drugs are in clinical use or under developmental process for tumor suppression: human or humanized mAbs targeting the immunosuppressive receptor PD-L1, such as atezolizumab, DVL, and avelumab. They are approved by FDA for the treatment of different cancer types like melanoma, non-small-cell lung cancer, renal cell carcinoma, urothelial carcinoma, liver cancer, and colorectal cancer.^19^ PD-L1 expression on tumor cells can be considered a predictive marker of increased invasion and invasiveness, but several studies are ongoing to clarify the mechanism of action of PD-L1 and its associated pathways. Since HER2-positive tumors and TNBC express high levels of PD-L1 and highly PD-L1-positive tumors are co-infiltrating with PD1+ infiltrating lymphocytes, a new therapeutic approach has been applied to combine anti-HER2 with novel anti-PD-L1 mAbs. It may also include combinations of EGFR drugs. Atezolizumab as monotherapy was tested in a phase I clinical trial with a cohort of 116 severely pretreated metastatic TNBC patients and was found to be effective and well tolerated.^18^ Loibl et al. denoted improved long-term outcomes in a randomized phase II trial, investigating the use of DVL in addition to anthracycline/taxane-based neoadjuvant chemotherapy in patients with early-diagnosed TNBC.^20^ According to a report, the response of TNBC patients to PD-1 inhibitors was relatively moderate (19%).^21^ This means that new treatments or combination therapies are needed to improve the effect of TNBC. Accumulating evidence from multiple trials indicated that the combination of immune checkpoint inhibitors and other anticancer agents in TNBC has achieved more significant results compared to monotherapy.^22,23^ As an innovative and successful drug, DVL used in TNBC patients is effective in inactivating this signaling pathway by binding to PD-L1, preventing PD-L1 from binding to PD-1. Hence, to the best of our knowledge, there have been no comprehensive reports in the literature on the influence of process variables on the cell-specific attachment of HSA-NPs by considering DVL targeting conditions.

## 2 Experimental Section

### 2.1 Materials

*N*-Hydroxysuccinimide (NHS), 1-ethyl-3-[3-(dimethylamino)-propyl] carbodiimide (EDC), 3-(4,5-dimethylthiazol-2-yl)-2,5-diphenyl-tetrazolium bromide (MTT), and human serum albumin (HSA) as lyophilized powder were purchased from Sigma-Aldrich (USA). DTX was received as a gift from DokuzEylül University Oncology Department in Turkey. Glutaraldehyde and all other chemicals were purchased from Merck. The cell culture medium DMEM, 1% l-glutamine, 10% FBS, penicillin (100 IU/mL), and streptomycin (100 IU/mL) were purchased from Gibco. Ultrapurified water was used during the analysis. The cells were purchased from ATCC.

### 2.2 Methodology, Preparation, and Characterization of PEGylated, DVL-Targeted, and DTX-Loaded HSA NPs

#### 2.2.1 Preparation of HSA NPs via Synthesizing the HSA-DTX Complex

HSA-DTX was synthesized by the desolvation method with modification of the described method^11,24^ and was dissolved in NaCl solution (10 mM, 1 mL). DTX (250 μg/12.5 μL) was dissolved in ethanol (62.5 μL) and added to the HSA solution. The pH of the solution mixture was adjusted to 8.5–9 with NaOH (0.5 M) solution. The solution was mixed with a stirring speed of 1400 rpm. Ethanol (3 mg) was added dropwise to the HSA-DTX solution at a rate of 1 mL/1 min. After stirring for 10 min at 1400 rpm, the speed was reduced to 500 rpm, and 10 μL of 8% glutaraldehyde solution was added. The total incubation time was 24 h. The solution was incubated for 8 h at a stirring speed of 500 rpm. Then, the solution was incubated at +4 °C for 16 h without stirring. After incubation, the supernatant was separated by centrifugation at 10,000 rpm. Two more washes were performed by adding the same volume (4 mL) of distilled water as the total amount of synthesis. Quality control was determined by dynamic light scattering (DLS), zeta potential, SEM, and Fourier transform infrared (FTIR) spectroscopy analyses.^25^

#### 2.2.2 Surface Modification by PEGylation of HSA-DTX NPs (HSA-DTX@PEG Synthesis)

The first surface modification of HSA-NPs was made with poly(ethylene glycol) 2-aminoethyl ether acetic acid (NH_2_-PEG5000-COOH). There are amine and carboxyl functional groups on the surface of albumins for surface modification. These groups provide targeting capability and unique features.^24^ The reaction is the activation of the carboxyl group (−COOH) of PEG by the EDC–NHS reaction and its binding to the amide group (NH_2_) on the surface of the HSA-NPs.^26^ 20 mg of PEG was weighed, dissolved in 1 mL of ultrapure water, and mixed at 500 rpm at room temperature. 96 mg of EDC [(1-ethyl-3-(3-dimethyl aminopropyl) carbodiimide hydrochloride)] (0.50 mmol) and 58 mg of NHS (0.50 mmol) were added equally in both reagents, dissolved in 750 μL of MES buffer each, and added to the PEG solution. The solution was mixed on a magnetic stirrer at 500 rpm for 30 min. Then, the pH was adjusted to 7 with a 2 M NaOH/HCI solution. After this activation step of PEG, 10 mg of HSA-DTX NPs was added slowly by dispersing them in 2.5 mL of distilled water. The mixture was stirred at 500 rpm for 4 h in the dark. After incubation, unreacted PEG was removed by centrifugation at 10,000 rpm for 30 min. The obtained synthesis product was washed by adding 5 mL of distilled water. The synthesized pellet was dispersed in 1 mL of distilled water, and after 5 min of homogenization, 10 μL of the sample was taken for size analysis. The centrifugation was repeated and dried by lyophilization.^24,27,28^ The obtained HSA-DTX@PEG NP was stored at 20 °C until used in the next step.

#### 2.2.3 Binding of DVL to HSA-DTX@PEG NPs (HSA-DTX@PEG-DVL)

DVL is activated for it to bind to the HSA-DTX@PEG NPs. For this, the EDC–NHS procedure has been applied. 10 μL (50 μg/μL) of DVL stock solution (5 mg/mL) was taken, and the volume was completed to 500 μL (10 mM) with 490 μL of phosphates-buffered saline (PBS). 1 mg/mL of the prepared EDC and NHS was dissolved in MES buffer with pH 4.7. 40 μL of EDC and 10 μL of NHS solutions were taken, and DVL solution was added while mixing in a magnetic stirrer (500 rpm). This solution was stirred at high speed at room temperature for 2 h 2.5 mg of HSA-DTX@PEG was added slowly to the medium by dispersion in 2.5 mL of PBS (pH = 7.2) and allowed to stir for 4 h. Afterward, it was incubated for 16 h at 4 °C without mixing. After centrifugation, the supernatant was separated and washed two times with the PBS buffer solution.^29^

#### 2.2.4 NP Characterization

The size distribution, hydrodynamic diameter, and zeta potential characterization of HSA-DTX, HSA-DTX@PEG, HSA-DTX@PEG-DVL, and HSA-DTX@PEG-DVL NPs were analyzed by the DLS method in a Malvern ZetaSizer device. All these measurements were carried out at Ege University, Institute of Nuclear Sciences Laboratory. The obtained size results in DLS measurements were compared by taking scanning electron microscopy (SEM) images for control purposes. Nanoparticle morphologies were determined with SEM. SEM analysis was performed at the Ege University Matal Laboratory. To confirm the conjugation of the HSA NPs, the chemical structure of the NPs was analyzed by FTIR spectroscopy.

##### 2.2.4.1 Determination of Drug Loading Efficiency of DTX-Loaded Nanoplatforms

High-pressure liquid chromatography analysis (HPLC Shimadzu with an LC-10Atvp quadruple pump, an SPD-10AV UV detector, and an FRC-10A fractionation collector) was performed to determine the loading efficiency of DTX. The loading efficiency of the amount of DTX in the synthesized HSA NPs can be calculated by directly determining the amount of drug contained in the NP or indirectly by determining the amount of drug in the free form. DTX standard solutions were prepared in concentrations of 250, 125, 62.5, and 31.25 μg/1 mL to determine the drug loading efficiency. The areas of the peaks formed at different concentrations were calculated by the HPLC method. A graph is drawn on which the calibration curve is constructed between the areas and the concentrations. The curve was used to determine the amount of drug, and HPLC analysis was performed by passing the stored samples through a 0.45 μm filter after washing. Our HPLC study was programmed according to the conditions applied by Rao et al.^30^ In the HPLC method, a C18 column was used, and water (solvent A) and acetonitrile (solvent B) were used as mobile phases. The mobile flow system is programmed as a gradient (Rao et al., 2006) in the first 15 min from the system, 65% A, 35% B; 15–20 min, 35% A, 65% B; 25–30 min, 25% A, 75% B; 30–35 min, 5% A, 95% B; and 35–40 min. The gradient system was arranged at 100% B per minute, and the wavelength in the UV detector was adjusted to 230 nm. The temperature was set at 25 °C, and the flow rate was 1 mL/min. The peak areas obtained after the injection of the prepared solutions into the HPLC column were plotted against the concentrations of the solutions, and the equation of the calibration curve was found by linear regression. The amount of free DTX in the upper phase was determined by using the equation obtained from the calibration curve. The loading efficiency (% LE) and loading capacity (% LC) of DTX were calculated using the following equations.
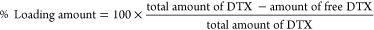

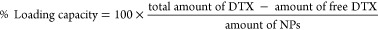


#### 2.2.5 In Vitro Drug Release Study

In this study, the release test of the chemotherapy drug DTX from the HSA-DTX@PEG-DVL NP was performed in a pH = 5.4 and 7.4 PBS environment. In the assay, 0.5 mg of HSA-DTX@PEG-DVL was dissolved in 2.5 mL of PBS. It was stirred in a water bath at 37 °C at 120 rpm for 72 h. Samples (500 μL) were taken at certain time intervals (30 min 1st, 2nd, 4th, 6th, and 24th; 48; and 72 h) and centrifuged at 10,000 rpm for 10 min. PBS solution was added to the medium as much as the sample is taken. Analysis of samples was carried out in HPLC. The change in the amount of DTX release over 72 h was determined by plotting the percent drug release graph of the samples taken at certain time intervals.

#### 2.2.6 Determination of Stability

The stability of HSA-DTX and HSA-DTX@PEG-DVL NPs was evaluated by determining the size change and zeta potential in the Malvern DLS-Zeta Sizer device.

#### 2.2.7 Detection of In Vitro Cytotoxicity, Post-Treatment Apoptosis/Necrosis Ratios of the HSA-DTX@PEG-DVL Nanoplatform in TNBC Cell Lines

##### 2.2.7.1 In Vitro Cytotoxicity Assays

MDA-MB-231, MDA-MB-468, and MCF-7 cell lines were cultured in DMEM F12 medium containing 10% FBS, 1% l-glutamine, and 1% penicillin–streptomycin. Cells were incubated at 37 °C in a 5% CO_2_ condition. After the cells reached 80% confluency, they were multiplied by passaging to 96-well plates, with 1000 cells in each well. After reaching confluency in wells, the MTT test was performed. By the MTT [3-(4,5-dimethyl-2-thiazolyl)-2,5-diphenyl-2*H*-tetrazolium bromide] colorimetric method and in vitro, cytotoxicity tests of DTX, HSA-DTX, and HSA-DTX@PEG-DVL nanoplatforms were performed. The IC_50_ dose of the agents was determined. MDA-MB-231, MDA-MB-468, and MCF-7 cell lines were seeded in 96-well cell plates with 10^4^ cells per well in 200 μL volume. The supernatants of the cells that adhered to 80–85% confluency the next day were removed from the wells, and DTX, HSA-DTX, and HSA-DTX@PEG-DVL nanoplatforms were applied in a volume of 100 μL. Different DTX concentrations of each of the nanoplatforms (30, 15, 7.50, 3.75, 1.88, and 0.94 μM) were added and incubated at 37 °C incubators for 24, 48, and 72 h. At the end of 24, 48, and 72 h, 10 μL of MTT (0.5 mg/mL) was applied to the wells. Plates were incubated at 37 °C for 4 h. After 4 h, each of the wells containing MTT was completely withdrawn, and 100 μL of DMSO was added to each well to dissolve the formazan crystals in the MTT. After keeping the plates in a 37 °C incubator for 15 min, the absorbance reading was taken at 570 nm wavelength. The percent cell viability was calculated by comparing control wells.

##### 2.2.7.2 Determination of Apoptosis/Necrosis Ratios after Treatment

To reveal the apoptotic effect of the nanoplatforms, Annexin V staining was performed and determined by the flow cytometry method. Propidium iodide (PI) dye was used to determine the rate of necrosis in cells. For this purpose, MDA-MB-231, MDA-MB-468, and MCF-7 cells were seeded in six-well plates. After 80% confluency, the cells were treated with LD50 doses determined by MTT tests for the appropriate time. LD50 doses at 48 h were used for MDA-MB-468, MDA-MB-231, and MCF-7 cells. While the effective dose of DTX was 15 μg in MDA-MB-468 and MDA-MB-236 cell lines, it was administered as 30 μg for MCF-7. For the MDA-MB-468 and MCF-7 cell lines, the effective dose of HSA-DTX was 15 μg, while the effective dose for the MDA-MB-236 cell line was 3.75 μg. The effective dose of HSA-DTX@PEG-MAB (7.5 μg) was administered to the MDA-MB-468, MDA-MB-231, and MCF-7 cell lines. The cells were then scraped off the plate and centrifuged. The resulting pellets were washed twice with cold PBS and dissolved in 1 mL of 1X Annexin Binding buffer. Separate tubes were coded for each condition. 100 μL (10^5^ cells) of thawed cells was transferred to tubes. In addition to the application of Annexin V and PI dyes to each condition, 100 μL of cells was transferred to unstained (UNS) tubes. Except for UNS tubes, 5 μL of Annexin V and 5 μL of PI dye were added to the cells. All tubes were vortexed and incubated for 15 min at room temperature. 400 μL of Annexin binding buffer was added to all tubes and analyzed on a BD Accuri C6 flow cytometer.

#### 2.2.8 ELISA Assay

The protocol for human PD-L1 ELISA was performed as an assay procedure. Briefly, standard dilutions were prepared (10, 5, 2.5, 1.25, 0.63, 0.32, 0.16 ng/mL, and blank). After that, samples were prepared (HSA-DTX@PEG-DVL (35 μg/ mL), 1:1 diluted HSA-DTX@PEG-DVL, MCF-7 supernatant, MCF-7 cell lysate, MDA-MB-231 supernatant, and MDA-MB-231 cell lysate). Then, 100 μL of standard dilutions was added into the appropriate wells in duplicate. Then, the samples were added to the appropriate wells. The plate was covered with a sealer, and the well plate was incubated for 90 min at 37 °C. After that, the solution was decanted carefully, and 100 μL of biotinylated detection Ab working solution was added to each well. The plate was covered with a sealer, and the well plate was incubated for 60 min at 37 °C. The solution was decanted carefully, and 350 μL of wash buffer was added to each well and soaked for 1 min. The solution was decanted carefully and dried against clean absorbent paper. This step was repeated three times. 100 μL of HRP conjugate working solution was added to each well. The plate was covered with a sealer, and the well plate was incubated for 30 min at 37 °C. The solution was decanted carefully, and the washing step was performed five times as conducted in previous steps. 90 μL of the substrate reagent was added to each well. The plate was covered with a sealer, and the well plate was incubated for about 15 min at 37 °C. During this time, the well plate was protected from light. After that, 50 μL of stop solution was added to each well. Immediately, the optical density (OD value) of each well was determined with a microplate reader set to 450 nm. The criteria for the analytical assay validation were performed following the current recommendations for bioanalytical methods: linearity, accuracy, precision, and reproducibility.

#### 2.2.9 DAPI Staining Assay

4′,6-Diamidino-2-phenylindole (DAPI) staining was performed to understand whether the HSA-DTX@PEG-DVL nanoplatform binds to the breast cancer cell’s DNA at the cellular level. and to demonstrate the apoptotic bodies formed in cells that were treated with the HSA-DTX@PEG-DVL nanoplatform. To perform this test, cells were first seeded in six-well plates. The next day, after the cells were attached to the surface of the plate, 2 mL of the HSA-DTX@PEG-DVL nanoplatform was applied to the cells. After 24 h, the medium was discarded carefully, and the cells were washed one to three times in PBS as needed. Then, sufficient 300 nM DAPI stain solution was added to cover the cells. The cells were incubated for 1–5 min and protected from light. After 5 min, the stain solution was removed. The cells were washed two to three times in PBS. The cells were imaged using a fluorescent microscope.

## 3 Results

### 3.1 Determination of Drug Loading Efficiency of the DTX-Loaded Nanoplatform

**Figure 1:**
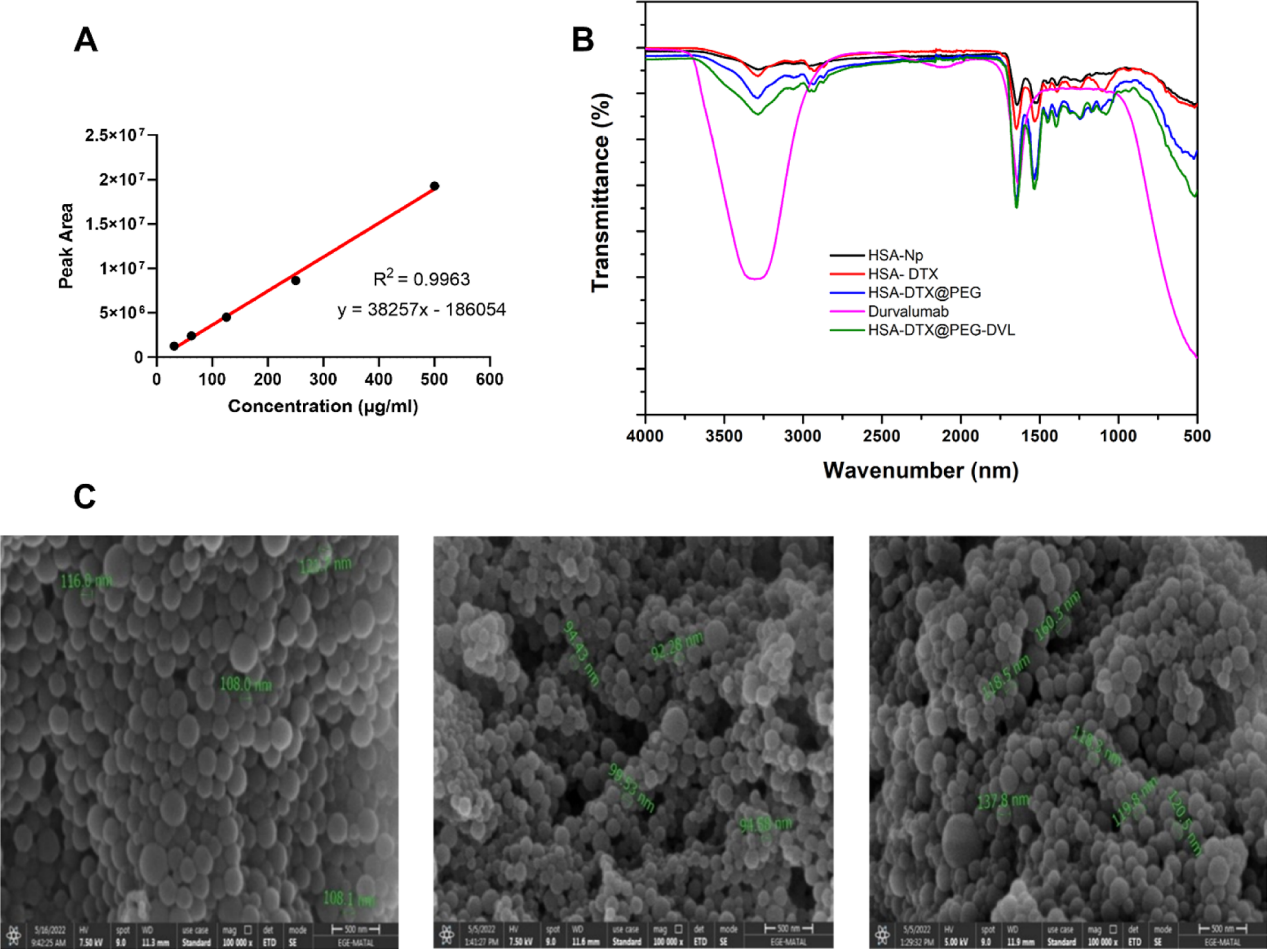
Calibration curve of various DTX concentrations (A). FT-IR spectra of HSA-DTX, HSA-DTX@PEG, HSA-DTX@PEG-DVL, and DVL (B). SEM images and DLS measurement results of HSA-DTX, HSA-DTX@PEG, and HSA-DTX@PEG-DVL nanoplatforms. The nanoparticles indicated within green circles in the SEM images represent the average size of each nanoparticle (C).

The HSA-DTX NP was prepared according to the desolvation method. To determine the percentage of DTX loading on HSA-DTX@PEG-DVL, HSA-DTX nanoplatform, UV absorptions of DTX solutions prepared at different concentrations were read in the HPLC device. A graph was drawn between concentration and absorbance. Unloaded DTX was determined by reading the absorption of the upper phase, and the amount of loaded DTX was determined as a result of the calculation. In the study of Rao et al., the DTX retention time was reported as 14.4 min (Rt: 14.4).^30^ In our study, the retention time of DTX was determined as 17 min (Rt: 17) under the same conditions. The area of this peak obtained in the HPLC analysis of the prepared solutions was plotted against the concentrations of the solutions, the calibration graph was drawn, and the equation of this linear graph was determined. The calibration chart and equation to be used in the quantification of DTX by HPLC are given in Figure 1A.

The amount of substance in the upper phase was determined by using the equation obtained from the calibration chart. The amount of material in the upper phase of the HSA-DTX@PEG-DVL nanoplatform was determined by the HPLC analysis and the amount of DTX that could not be loaded. The DTX drug loading efficiency was determined to be 91.8 ± 3.9%, and the drug loading capacity was determined to be 2.2 ± 1%. Kordezangeneh et al. found the loading efficiency of HSA-DTX to be 98 ± 1.4% and the loading capacity to be 0.98 ± 0.01%.^31^

### 3.2 Characterization of the Synthesized Nanoplatform

#### 3.2.1 FTIR Spectroscopy of HSA-DTX

The FTIR spectra of HSA and HSA-DTX are shown in Figure 1B. As can be seen from the spectrum, when the spectra of HSA and HSA-DTX were compared, not much difference was observed between the spectra. The amide-I band in proteins is mainly formed at 1600–1700 cm^–1^ due to the C=O stretching vibrations in the amide groups. The amide-II band is mainly located in the C–N stretch pair region with the *N*–H bending pattern at 1500–1600 cm^–1^. The amide-I band is more sensitive to changes in the secondary structure of the protein than the amide-II band.^32−34^ In the study of Chen et al., it was observed that amide-I and amide-II peaks shifted at 1644 and 1531 cm^–1^, respectively. It was determined that amide-I and amide-II absorptions increased with the addition of DTX, and the width of this peak increased. This indicates that the secondary structure of the protein has been changed. DTX interacts with the −C=O and −C–N groups in protein polypeptides and causes rearrangement of the polypeptide carbonyl hydrogen bond network. The peak of the −OH group of HSA-DTX can be seen at 3280 cm^–1^. The carboxyl groups in the PEG structure were conjugated to NH_2_-HSA over the reaction with the carbimide environment. Different peaks were observed in the spectrum of PEG-COOH and HSA-NH_2_. The amide-I band in proteins mainly occurs at 1600–1700 cm^–1^ due to the change in amide groups due to the C=O stretching vibrations. The amide-II band (1650–1515 cm^–1^), a weak C–N stretching band (1307–1395 cm^–1^), can be seen in amides 1650 and 1533 cm^–1^ due to the NH bond containing C–N stretching vibration. It was determined that the HSA-DTX@PEG NP C–O tensile band shifted at 1038–1168 cm^–1^ at 1083 cm^–1^, which corresponds to the stretches in the C–O band in HSA-DTX NP (Figure 1B). It confirms that it reacts with two broad peaks detected at 1648 and 1533 cm^–1^ with minimal differences in the peak region where the amide bonds are located compared to HSA-DTX NP and a shift in the peaks located between 1400 and 1750 cm^–1^ in the PEG chain.^35^ HN–C=O, the peak was detected at 2165 cm^–1^. In addition, the −OH band of the carboxyl group is observed at 3200 cm^–1^.^35−37^ In the synthesis of HSA-DTX@PEG-DVL, DVL binding occurs with the same reaction. The wide peak between 1650 and 1533 cm^–1^, which shows that the EDC–NHS reaction has been successful, expresses the C=O–NH stretch band and the COO-carboxylate.^38^ Compared to DVL’s peak in the spectrum of HSA-DTX@PEG-DVL in Figure 1B, the energy from 1634 cm^–1^ slightly reflected the peak at 1646 cm^–1^ and changed the intensity of the peak, so there are overlapping peaks in the structure.^39^

#### 3.2.2 Dimensional Analysis Data of HSA-DTX, HSA-DTX@PEG, and HSA-DTX@PEG-DVL

The size and zeta potential characterization of the HSA-DTX, HSA-DTX@PEG, and HSA-DTX@PEG-DVL nanoplatforms were performed using the DLS method on the Malvern ZetaSizer device (Table 1).

**Table 1:** DLS and Zeta Measurement Results of Nanoplatforms

	*Z*-average (nm)	PDI	zeta potential (mV)
HSA-DTX	168 ± 5	0.277	–31.3 ± 2
HSA-DTX@PEG	130.4 ± 4	0.526	–30.1 ± 1
HSA-DTX@PEG-DVL	178.1 ± 5	0.310	–31.9 ± 3

Ultrapure water was used as the solvent while determining the particle sizes and zeta potentials. As a result of the DLS and zeta potential measurements of the HSA-DTX NP, the hydrodynamic radius was determined to be 168 ± 5 nm and the zeta potential as −31.3 ± 2 mV. In the study conducted by Kordezangeneh et al., it was determined that the NP size was 146 nm, and the zeta potential was −29 mV.^31^ It is stated that the negative zeta potential is due to the carboxyl groups found in HSA.^31^ The high negative zeta potential value indicates that the NPs are stable. Jiang et al. also stated that the particle size is between 120 and 160 nm in HSA-DTX synthesis.^25^ The NP size and zeta potential are also in agreement with the literature. The hydrodynamic radius of the HSA-DTX@PEG NP is 130.4 ± 4 nm, the zeta potential is −30.1 ± 1 mV, while the hydrodynamic radius of the HSA-DTX@PEG-DVL NP is 178.1 ± 5 nm, and the zeta potential is −31.9 ± 3 mV. These values represent the mean values from at least three synthesis replicates.

#### 3.2.3 Scanning Electron Microscopy

The images of HSA-DTX, HSA-DTX@PEG, and HSA-DTX@PEG-DVL nanoparticles determined by SEM analysis are given in Figure 1C. The nanoparticles were spherical and homogeneously dispersed. Dimensions were obtained from SEM analysis images, and DLS data were compared. In the SEM analysis results, it was observed that the NP sizes were smaller in their images. According to the literature, the sizes of HSA NPs varied in the range of 100–200 nm.^11^ In the results of SEM analysis, it was observed that the sizes of HSA-DTX NPs changed in the range of 108 ± 5 nm. The dimensions of the HSA-DTX@PEG NPs changed by 93 ± 2 nm. It varied in the 128 ± 30 nm range of HSA-DTX@PEG-DVL NPs. The morphologies of HSA-DTX, HSA-DTX@PEG, and HSA-DTX@PEG-DVL NPs were determined to be spherical. In the coating of NPs, a surface coating process of 5–15 nm thickness containing 80% Au and 20% Pd was carried out.

#### 3.2.4 UV Spectra of the HSA-DTX@PEG-DVL Nanoplatform Synthesis Intermediate

UV–vis spectrum measurement is a method for demonstrating structural change; the UV–vis absorption spectra of HSA-DTX, HSA-DTX@PEG, and HSA-DTX@PEG-DVL are shown in Figures S1–S3, respectively. HSA-DTX HSA shows a strong absorbance at 205 nm due to the α-helical structure of the protein. In the spectrum of HSA-DTX-bound PEG and DVL-bound NPs, it can be seen that this absorbance peak decreases. In Figure S1, the UV absorption wavelength of the HSA-DTX@PEG NP is 203 nm, while the wavelength of the HSA-DTX@PEG-DVL NP is 200 nm. As a result, it has been observed that each surface modification made on HSA-DTX NPs causes shifts in the absorption value of the peak in HSA-DTX NPs, and these findings are in agreement with the literature.^40^ Thus, it can be seen that the HSA-DTX@PEG-DVL nanoplatform has been successfully synthesized.

### 3.3 In Vitro Drug Release

**Figure 2:**
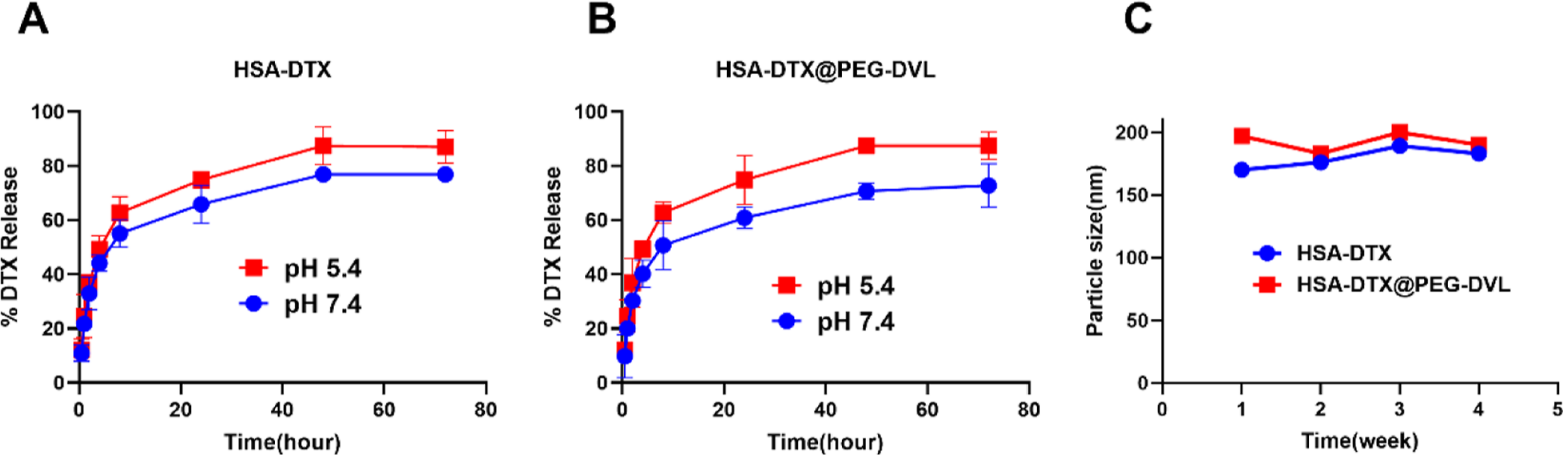
Release graph of DTX from the HSA-DTX nanoplatform (A). Release graph of DTX from the HSA-DTX@PEG-DVL nanoplatform (B). Stability of HSA-DTX and HSA-DTX@PEG-DVL nanoplatforms (C).

Drug release systems have become a promising area today due to their targeting properties and adjusting dose of active substances to an adequate amount thanks to controlled release compared to conventional drug forms.^41^ The HSA-DTX@PEG-DVL and HSA-DTX release assay was performed in PBS pH 7.4 and PBS pH 5.4 release buffers. The difference in drug release activity in cancer cells and normal cells was investigated by the release assay performed in PBS buffers at different pHs. The graphs plotted with the data obtained because of the pH 5.4 and pH 7.4 release tests of the HSA-DTX and HSA-DTX@PEG-DVL NPs are shown in Figure 2A,B. Results are presented as a percentage of cumulative release over 72 h. A faster release of HSA-DTX@PEG-DVL NP and HSA-DTX NP was observed at pH 5.4 compared to pH 7.4 in a PBS medium.^42^ It shows that in the case of a PBS medium at pH 7.4, there may be less drug release and fewer side effects during blood circulation.^43^ In HSA-DTX@PEG-DVL NP pH 7.4, 50% drug release was observed in PBS medium after 8 h, while 50% drug release was observed after 4 h at pH 5.4. In the release test performed in PBS medium for pH 7.4, 73% of the drug was released in 72 h, while in the experiment performed in PBS medium for pH 5.4, approximately 87% of the drug was released after 72 h. The HSA-DTX NP released 49% drugs after 4 h at pH 5.4. At the end of 72 h, 87% of the drug was released. At pH 7.4, it released 55% of the drug after 8 h. After 72 h, 76% of the drug was released. This release behavior indicated that the drugs would be released at lower pH, that is, at tumor sites. A controlled release of DTX was observed from the HSA-DTX@PEG-DVL nanoplatforms.

### 3.4 Stability

The stability of HSA-DTX and HSA-DTX@PEG-DVL NPs was evaluated by looking at the change in size and zeta potentials over 4 weeks, and the structural integrity of the nanoparticle was evaluated (Figure 2C). Small changes in the standard deviation range in the size of the NPs indicated that the NPs were stable. Abolhassani and Shojaosadati found that HSA-NPs obtained by the desolvation method were stabilized with glutaraldehyde and therefore had higher stability and therefore more negative charge.^44^ The NPs prepared by the desolvation method, which we also applied in our study, showed high stability due to their high negative charges and regular size distributions.

The potential of PD-1/PD-L1 pathway inhibition as a therapeutic strategy has been studied in various cancer types. However, there is a requirement for tumor-targeted delivery systems for ensuring safety since PD-L1 is also expressed in normal tissues and cells.^45^ In this study, we have developed a strategy based on DVL-targeted and DTX-loaded HSA-NP (HSA-DTX@PEG-DVL NP) synthesis to induce the membrane surface expression of PD-L1 in TNBC cancer cells, providing higher sensitivity, superior drug uptake, and wide cell apoptosis. Our data indicate that this approach can be an effective way to enhance HSA-DTX@PEG-DVL NP cytotoxicity to TNBC cancer cells via targeting with DVL, considering that these cells lack membrane surface targeting receptors to perform immunoreactive therapy. In addition, DTX has demonstrated promising cell cytotoxic activity, which led to interference with cell division and caused cell death when the use of DTX is considered to exhibit hydrophobicity and degradation characteristics. Furthermore, DTX is a limited delivery characteristic type of drug due to poor water solubility, and it requires carrying the tumor site at an effective concentration.^46,47^ This study represents a new strategy for PD-L1-based targeting therapy for TNBC due to the specificity and effectiveness of the HSA-DTX@PEG-DVL NPs. In addition, the drug delivery HSA-NP system can offer targeted delivery of PD-L1 immunotherapeutics, which can make important contributions to tumor immunotherapy and offer us a new perspective to address the limitations mentioned above. The studies indicate that the HSA-NPs have excellent properties such as high biocompatibility, good biodegradability, and low toxicity; these features assist the particle in escaping from phagocytosis and opsonization and thus can be used as an effective and safe targeted drug carrier for PD-L1 inactivation.^6−11^ Another important property of HSA-NPs is their excellent ability to a quick release of the cancer drug which ensures effective TNBC cell inhibition.^48−51^ The use of the desolvation method to synthesize HSA-NP and layer-by-layer modification on this nanostructure presents good reproducibility, scalability, and size. Regarding stability, we found that the HSA-NPs are stable for at least 1 month at −20 °C. We have also shown that our DVL antibody-conjugated HSA-NP NPs can be lyophilized without losing their physical characteristics and efficacy over time. We conjugated DVL to the surface of HSA-NPs via EDC–NHS coupling agents, and it improves the delivery efficiency of DTX to the targeted site of the tumor and helps control the administration time and dosage. As a drug carrier, HSA-NPs can increase the solubility of hydrophobic drugs such as DTX and offer surface properties to extend blood circulation. In addition, it allows for their selective accumulation in tumors resulting from the EPR effect.^52^ By modifying the surface of drug-loaded HSA-NPs with DVL, tumor accumulation may be further enhanced and provided to overcome these therapeutic limitations because the non-specific interaction between the NP and non-target cells is a situation that highly affects the therapeutic efficacy resulting in adverse effects.

### 3.5 In Vitro Cytotoxicity of the HSA-DTX@PEG-DVL Nanoplatform Assay

**Figure 3:**
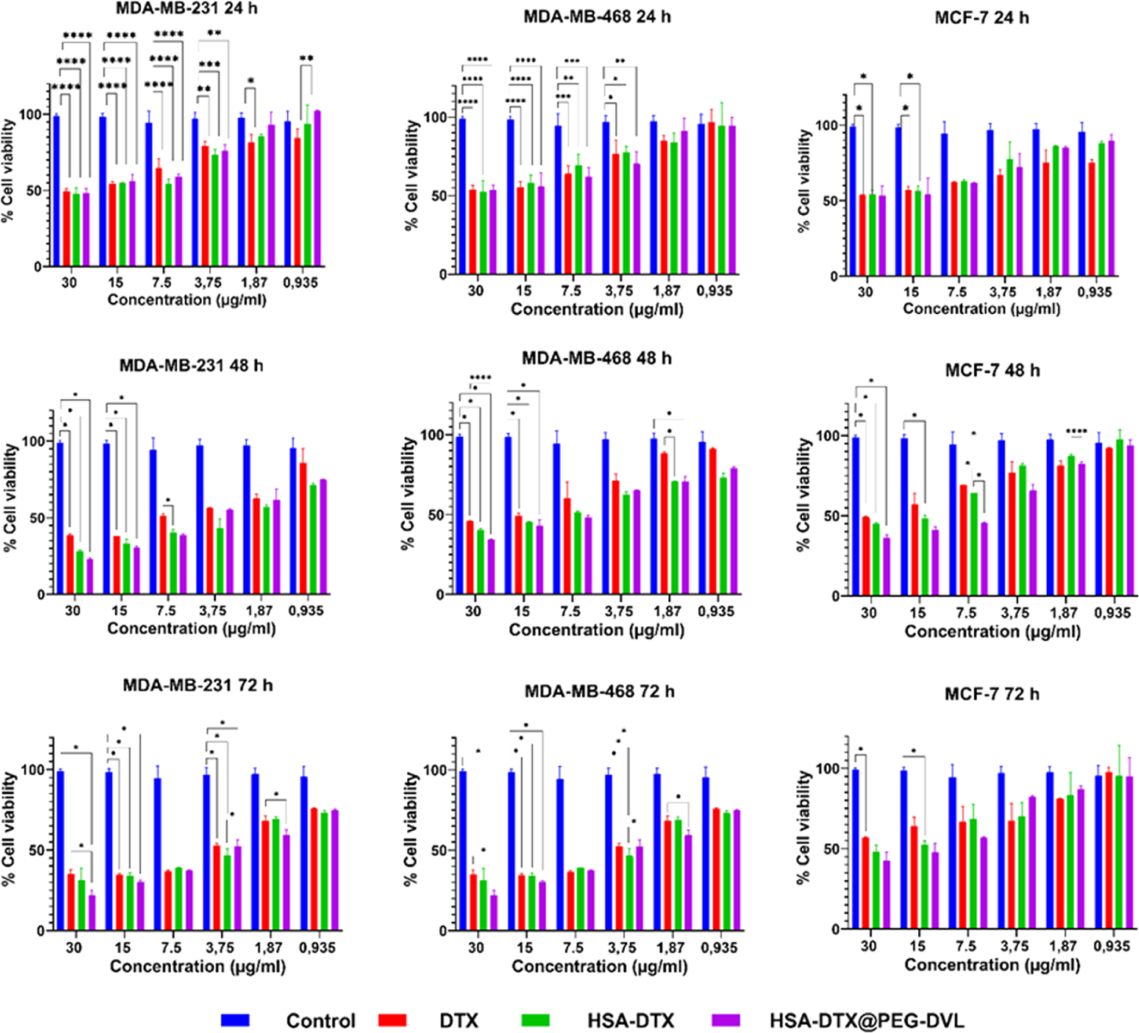
Cell viability results of the HSA-DTX@PEG-DVL nanoplatform on MDA-MB-231, MDA-MB-468, and MCF-7 cells. A two-way RM ANOVA was performed for statistical analysis of the cytotoxicity tests, followed by Tukey’s multiple comparisons test to test the *p*-values. The *p*-values were indicated in the corresponding areas in Figure 3, where **** represents *p* < 0.000001, *** represents *p* < 0.00001, ** represents *p* < 0.0001, * represents *p* < 0.001, and ns represents *p* < 0.01. The cytotoxicity tests were repeated three times, with four replicates per experiment (*n* = 4) in each trial.

MTT analysis was performed at 24, 48, and 72 h for each cell line in 96-well plates. As indicated in the graph of the viability of the MTT analysis performed after 24 h in Figure 3, none of the agents had a more than 50% lethal effect on the cells. The doses at which we found the IC_50_ dose to be effective were seen in 48 h, and for the TNBC cell line MDA-MB-468, DTX was determined as 15 μg, HSA-DTX 15 μg, and HSA-DTX@PEG-DVL 7.5 μg. For another cell line, MDA-MB-231, DTX is 30 μg, HSA-DTX is 3.75 μg, and HSA-DTX@PEG-DVL is 7.5 μg. For the hormone receptor-positive cell line MCF-7, DTX was 30 μg, HSA-DTX 15 μg, and HSA-DTX@PEG-DVL 7.5 μg. In Figure 3, vitality values below 50% were observed in the MTT performed at 72 h. Since the same effective doses were seen at the 48th hour, the duration of activity was determined as 48 h. Kordezangeneh et al. also found the IC_50_ dose of HSA-DTX effective at 48 h in their study.^31^ The higher cytotoxicity displayed by HSA-DTX@PEG-DVL is due to the combined effect of DVL and DTX. In vitro cell viability results for breast cancer cells are consistent with the previously reported literature.^53^

HSA-DTX@PEG-DVL NP showed better cytotoxicity compared to DTX in all the three cell lines at different time points (24, 48, and 72 h) in both MDA-MB-231 and MCF-7. We observed higher IC_50_ values for free DTX. The higher IC_50_ values of DTX may be due to drug resistance. Results obtained with cell viability on various nanoplatforms loaded with DTX and DTX are in agreement with breast cell lines.^53,54^

### 3.6 Apoptosis/Necrosis Rates

**Figure 4:**
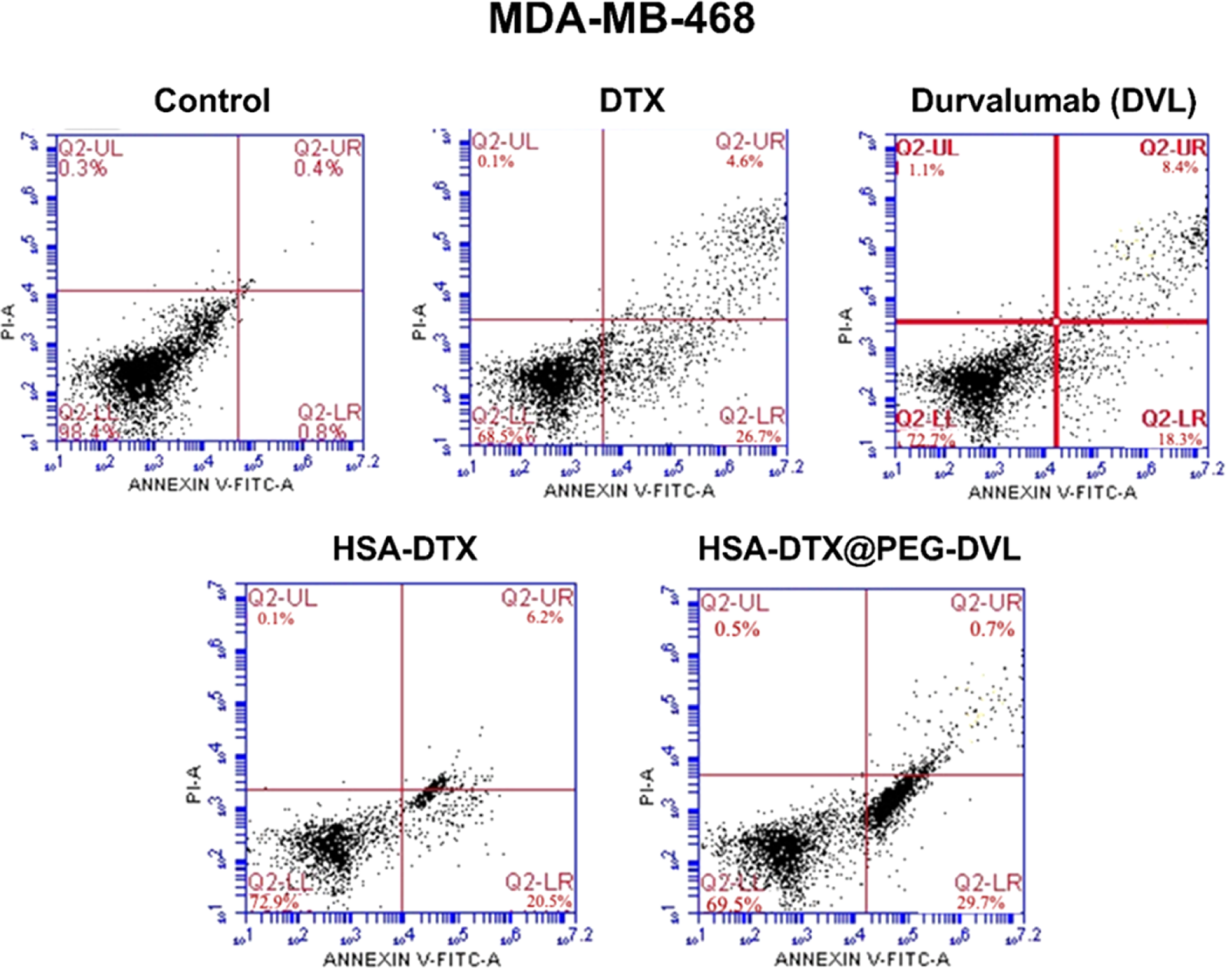
Flow cytometry results of HSA-DTX and HSA-DTX@PEG-DVL nanoplatforms, DTX, and DVL alone at MDA-MB-468 cells.

From staining with FITC Annexin V/PI to determine the apoptosis/necrosis rates, the necrosis rates of the agents were determined to be less than 5% in all the three cell lines. It was observed that the agents did not lead the cells to late apoptosis but were effective in the early apoptotic stage (Table 2). HSA-DTX led to more apoptosis than DTX given in the free form. DVL, which is known to be effective in a TNBC cell line, MDA-MB-468, has a higher apoptosis rate when targeted to the nanostructure than in the untargeted condition of the nanostructure (Figure 4 and Table 2).

**Table 2:** Apoptosis/Necrosis Rates for the MDA-MB-468 Cell Line

MDA-MB-468	% cell viability	% early apoptosis	% late apoptosis	% necrosis
control	94.7	3.2	0.0	2.1
DTX	68.5	26.7	4.6	0.1
HSA-DTX	72.9	20.5	6.2	0.1
HSA-DTX@PEG-DVL	69.5	29.7	0.7	0.5
DVL	72.2	18.3	8.4	1.1

**Figure 5:**
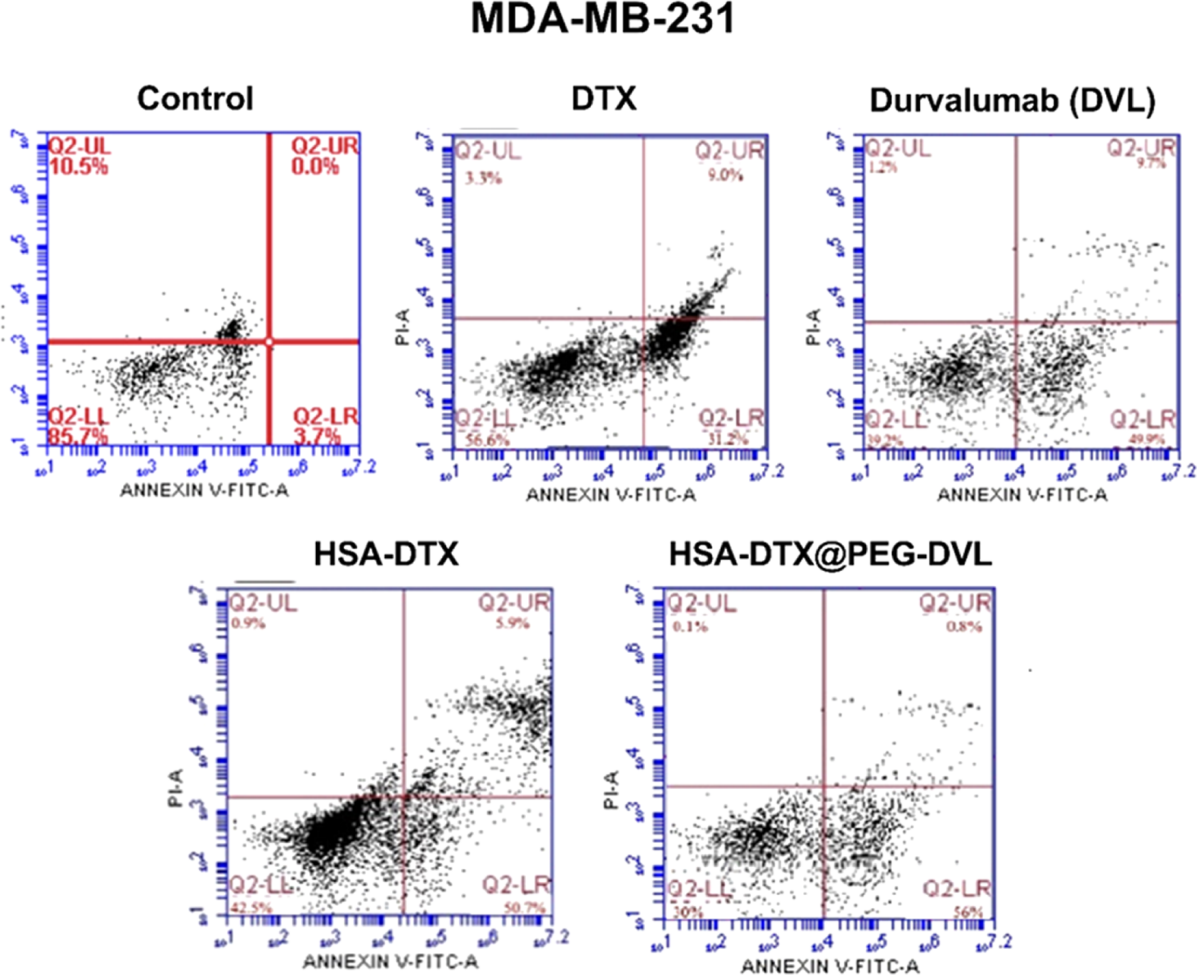
Flow cytometry results of HSA-DTX and HSA-DTX@PEG-DVL nanoplatforms, DTX, and DVL alone at MDA-MB-231 cells.

Although the percentage of total death was close to each other in all conditions, targeting the nanostructure with a mAb was found to reduce necrosis and increase apoptosis. MDA-MB-231 cells showed a 40% apoptosis rate in free DTX conditions. The other condition is that when the drug is loaded into the nanostructure, the HSA-DTX apoptosis rate was determined as 56.6%. Free DTX displayed a higher rate of necrosis than HSA-DTX. Accordingly, loading DTX into HSA is important in terms of dragging cells into necrosis. We observed the highest apoptosis rate in the HSA-DTX@PEG-DVL nanostructure, revealing that targeting the nanostructure with a mAb decreased the rate of necrosis and increased the rate of apoptosis (Figure 5).

**Figure 6:**
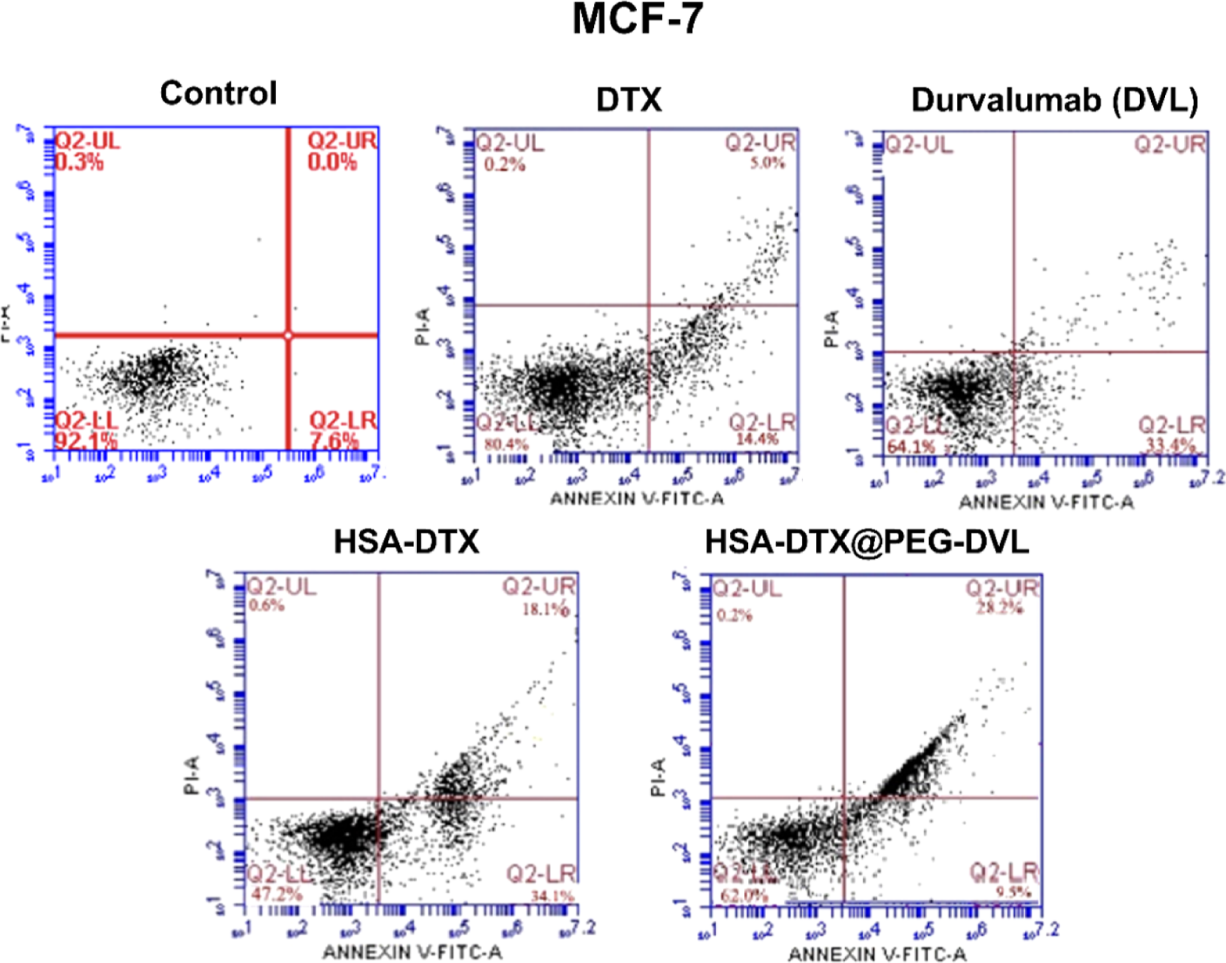
Flow cytometry results of HSA-DTX and HSA-DTX@PEG-DVL nanoplatforms, DTX, and DVL alone at MCF-7 cells.

Compared to free-DTX, HSA-DTX loaded into the nanostructure showed more cell death. In MCF-7 DTX cells, the percentage of total apoptosis was 19.4%. In HSA-DTX, this rate increased to 52%. The viability rate decreased compared to the free DTX. Accordingly, HSA-DTX led cells to apoptosis more than free-DTX (Figure 6). MCF-7 is a hormone receptor-positive cell line. Since DVL is an anti-PD-L1 agent, it affects TNBC due to the ability of TNBC cells to express PD-L1. However, the PD-L1 expression level is too weak in receptor-positive cells (Table 3).^55^

**Table 3:** Apoptosis/Necrosis Rates for the MDA-MB-231 Cell Line

MDA-MB-231	% cell viability	% early apoptosis	% late apoptosis	% necrosis
control	85.7	3.7	0.0	10.5
DTX	56.6	31.2	9.0	3.3
HSA-DTX	42.5	50.7	5.9	0.9
HSA-DTX@PEG-DVL	30.0	56.0	0.8	0.1
DVL	39.2	49.9	9.7	1.2

Therefore, the lethal activity of HSA-DTX@PEG-DVL was not observe in MCF-7 cells as much as in TNBC cells. MDA-MB-468 and MDA-MB-231 cells are both TNBC cell lines, and the HSA-DTX@PEG-DVL nanoplatform was found to be more effective in the MDA-MB-231 cell line. Mittendorf et al. showed that the PD-L1 surface expression is higher in MDA-MB-231 cells, and this expression is related to the PTEN pathway.^56^ In our study, MDA-MB-231 showed more apoptotic death than MDA-MB-468, which is confirmed in the literature (Table 4).

**Table 4:** Apoptosis/Necrosis Rates for the MCF-7 Cell Line

MCF-7	% cell vitality	% early apoptosis	% late apoptosis	% necrosis
control	92.1	7.6	0.0	0.3
DTX	80.4	14.4	5.0	0.2
HSA-DTX	47.2	34.1	18.1	0.6
HSA-DTX@PEG-DVL	62.1	9.5	28.2	0.2
DVL	64.1	33.4	1.4	1.2

### 3.7 ELISA Validation

**Figure 7:**
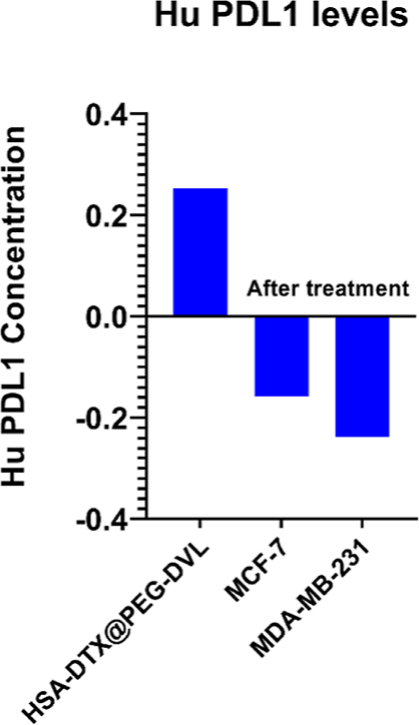
ELISA results of PD-L1 levels in HSA-DTX@PEG-DVL and breast cancer cell lines.

A Sandwich ELISA assay was performed to determine PDL-1 protein levels in the HSA-DTX@PEG-DVL nanoplatform. It was performed with the HSA-DTX@PEG-DVL nanoparticle to determine how much DVL is bound to the structure. Also, to assess potential HSA-DTX@PEG-DVL-induced changes in PD-L1 protein expression in breast cancer cells, MDA-MB-231 and MCF-7 cells were treated with the nanostructure form (7.5 μg/mL) for 24 h and analyzed. Accordingly, the results are as follows. Figure 7 shows a significant decrease in PD-L1-positive breast cancer cells and the amount of bonding to the nanostructure. As can be seen, DVL antibodies successfully bind to the nanostructure. HSA-DTX@PEG-DVL NP, which appears to be effective on cells, appears to completely suppress protein expression in MCF-7 and MDA-MB-231 cells. According to this result, nanoplatform is effective on breast cancer cells as a treatment agent.

### 3.8 DAPI Staining

**Figure 8:**
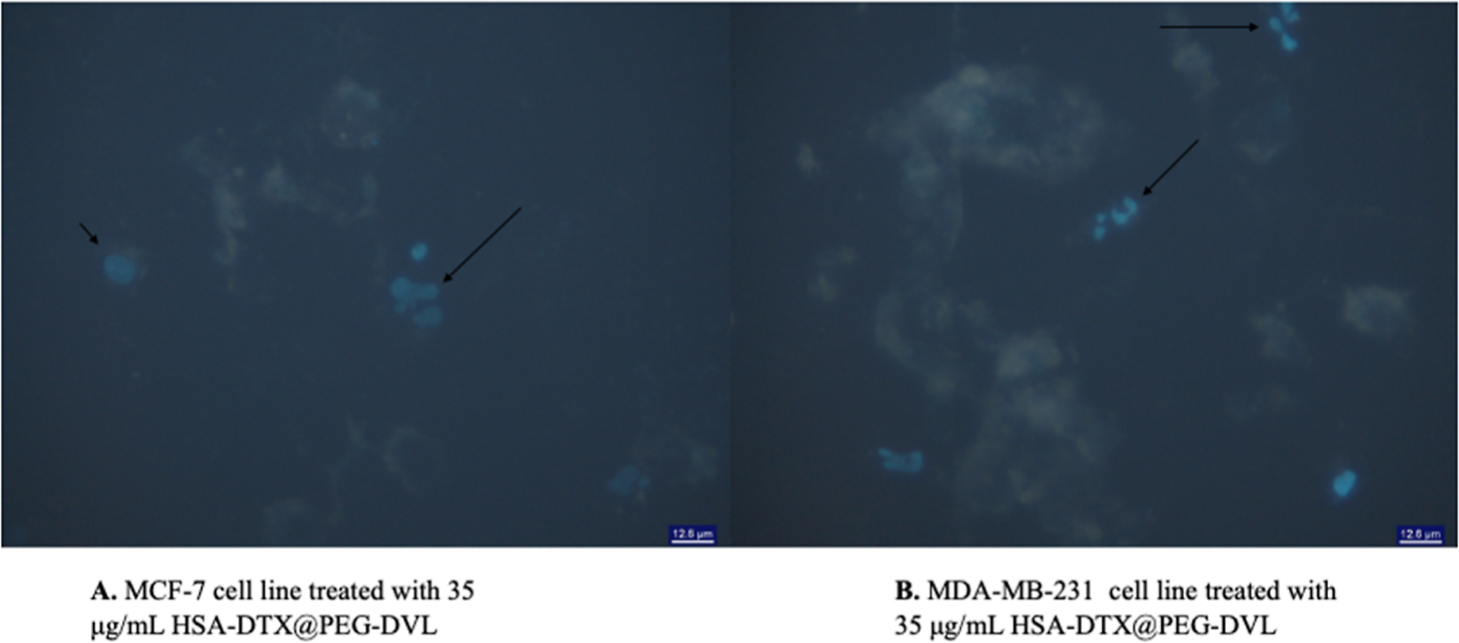
MCF-7 and MDA-MB-231 cells were treated with 35 μg/mL HSA-DTX@PEG-DVL for 24 h and stained with DAPI. Chromatin condensation, representing apoptotic cell death, was examined using a fluorescence microscope (x400). The short arrow indicates the living cell. The long arrows indicate apoptotic cells. Cells stained blue with DAPI are driven to apoptosis. Our findings show that apoptosis is induced by the treatment of cells with HSA-DTX@PEG-DVL. The percentage of late apoptotic (DAPI-positive) cells after 24 h of incubation was 35 ± 2% for MCF-7 and 60 ± 2% for MDA-MB-231, respectively. Our findings collected from DAPI staining correlate with the Annexin-V/PI apoptosis results. Accordingly, HSA-DTX@PEG-DVL shows an apoptotic lethal mechanism on TNBC cancer cells.

To evaluate the effect of the HSA-DTX@PEG-DVL nanoplatform on the nucleus, we performed DAPI staining. DAPI forms fluorescent complexes with double-stranded DNA and stained nuclei brightly fluoresce under a DAPI filter. As shown, MDA-MB-231 and MCF-7 cells treated with 7.5 μg/mL HSA-DTX@PEG-DVL fluoresced brightly, indicating chromatin condensation (Figure 8).

## 4 Discussion

The DVL-targeted HSA-NPs contributed to improving the cellular uptake and enhancing the drug cytotoxic efficiency in MDA-MB-231, MDA-MB-468, and MCF-7 cells. Furthermore, we evaluated the cell apoptotic levels of all intermediate forms of the HSA-NPs on these cell lines. The results indicate that the PD-L1 biomarker increases the tumor-targeting effects and led to high apoptotic levels in all the cells (Figures 4–6). In addition, new findings in the literature suggest that DTX may also increase the expression of PD-L1 in cancer cells, which means making target cells more sensitive to PD-L1 mAbs like DVL.^57^ According to the results, the HSA-DTX@PEG-DVL NPs effectively deliver DTX to tumor cells, causing a cytotoxic effect rate of up to 70% on both TNBC cells at 72 h and increasing cell apoptosis (Figure 3). The reason why for these results, the tumor cells may be more vulnerable to the DVL immune attack after exposure to DTX therapy.^58,59^ Results from ELISA showed that after treatment of the PD-L1 level on the MDA-MB-231 cells, the supernatant was higher than that of MCF-7 cells. The cellular uptake of HSA-DTX@PEG-DVL NPs showed that DTX can be enhanced to a certain concentration even in MCF-7 cells with low PD-L1 expression. In addition, using a low concentration of HSA-DTX@PEG-DVL NPs contributed to cell inhibition on these cell lines, and this result also indicates that the linked DVL has had biological activity. Moreover, according to our research, the inhibition might be caused by DVL and thus further increases the drug sensitivity of DTX. The advantages of our HSA-DTX@PEG-DVL NPs in terms of cytotoxicity might be mainly due to cellular uptake superiority and synergy between DVL and DTX, much of the literature also suggested that finding.^60−62^ Consequently, we have shown that the combination of the anticancer drugs DTX and DVL in a single nanoplatform significantly reduces tumor cell progression compared to each drug alone. As a result, our findings suggested that the addition of the DVL mAb on HSA-DTX@PEG-DVL NPs loaded with DTX is a better solution for anticancer efficacy. We believe that the development of surface chemistries of newly synthesized NPs will provide excellent opportunities to improve the efficacy of immunotherapy in the treatment of critical diseases such as TNBC.

## 5 Conclusions

In this study, we found that DVL targeted mediated knockdown of PD-L1 on TNBC cell lines and significantly enhances the cytotoxicity of the nanodrug formulation to cancer cells. These data suggest that a targeted strategy with DVL has been achieved in TNBC cell types. To overcome the dose-limiting current toxicity of DTX, we developed a DTX-loaded HSA-NPs and conjugated it to DVL to provide synergetic effects of PD-L1 blockade. The efficacy of HSA-DTX@PEG-DVL NPs in targeting TNBC tumors in vivo will be investigated in future studies. Moreover, for further studies, our strategy will be potentially an alternative for PD-1/PD-L1-targeted cancer nanotherapy.

## Supporting Information Available

UV spectra of HSA-DTX, HSA-DTX@PEG, and HSA-DTX@PEG-DVL nanoplatforms (PDF)
