# Supplementary material for: Synthesis and Optimization of the Docetaxel-Loaded and Durvalumab-Targeted Human Serum Albumin Nanoparticles, In Vitro Characterization on Triple-Negative Breast Cancer Cells
Source: ACS Omega. 2023 Jul 13;8(29):26287–300. doi: 10.1021/acsomega.3c02682 (PMC10372957; doi:10.1021/acsomega.3c02682)
Supplement: Supplementary file 1 — ao3c02682_si_001.pdf [file ao3c02682_si_001.pdf]

Supporting Information

Synthesis and optimization of the Docetaxel-loaded and Durvalumab-targeted human serum albumin nanoparticles, *in vitro* characterization on Triple-Negative Breast Cancer Cells

Fatma Yurt[1], Derya Özel[1], Ayça Tunçel[1], Ozde Gokbayrak[2], Safiye Aktas[2]*

[1]*Department of Nuclear Applications, Institute of Nuclear Science, Ege University Bornova, 35100, Izmir, Turkey.*

[2]*Department of Basic Oncology, Institute of Oncology, Dokuz Eylül University, Izmir, Turkey*

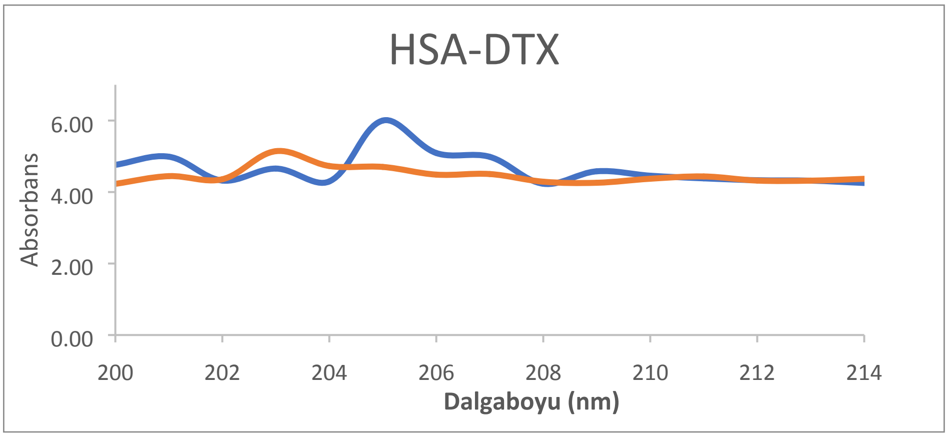

Figure S1. UV Spectra of HSA-DTX nanoparticle

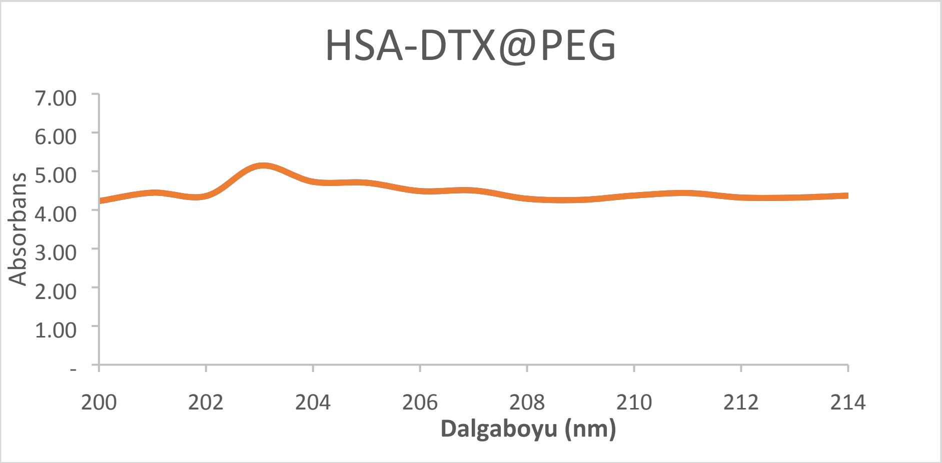

Figure S2. UV Spectra of HSA-DTX@PEG nanoplatform

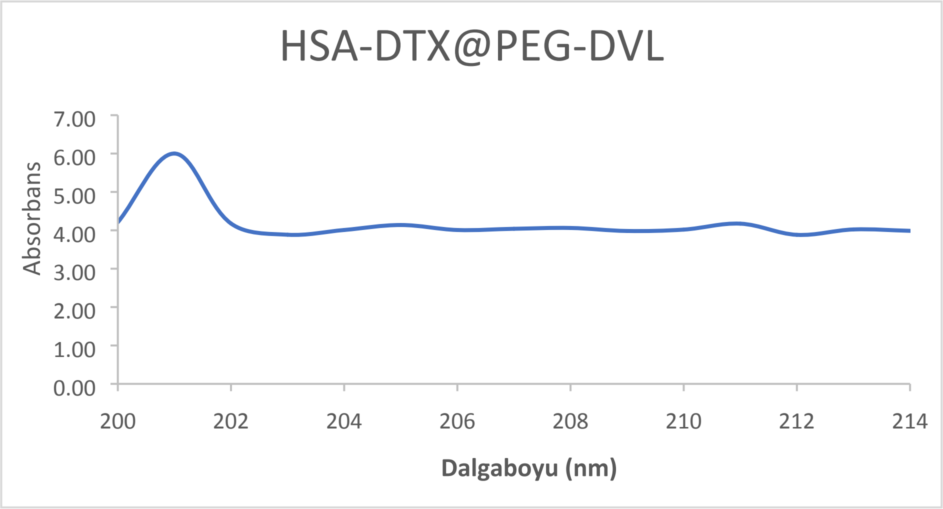

Figure S3. UV Spectra of HSA-DTX@PEG-DVL nanoplatform
